# Editorial: Links between regenerative medicine and immunotherapy: how cellular therapies modulate immune responses for improved outcomes

**DOI:** 10.3389/fvets.2024.1462218

**Published:** 2024-07-26

**Authors:** Lynn Pezzanite, Laura Barrachina, Lauren Schnabel

**Affiliations:** ^1^Translational Medicine Institute, Department of Clinical Sciences, College of Veterinary Medicine and Biomedical Sciences, Colorado State University, Fort Collins, CO, United States; ^2^Laboratorio de Genética Bioquímica LAGENBIO, Instituto de Investigación Sanitaria de Aragón (IIS), Instituto Agroalimentario de Aragón-IA2 (Universidad de Zaragoza-CITA), Universidad de Zaragoza, Zaragoza, Spain; ^3^Comparative Medicine Institute, Department of Clinical Sciences, College of Veterinary Medicine, North Carolina State University, Raleigh, NC, United States

**Keywords:** regenerative therapy, biologic therapy, mesenchymal stem cell, platelet lysate, immunotherapy

## Editorial summary

### Overview

We are pleased to serve as guest editors for this special Research Topic in *Veterinary Regenerative Medicine* highlighting how regenerative therapies modulate immune responses to elicit a therapeutic effect. The field of veterinary regenerative medicine has advanced considerably in the past two decades, with multiple treatment options marketed to veterinary clinicians (e.g., mesenchymal stromal cells or extracellular vesicles from various tissue sources, platelet derived products, autologous conditioned serum or protein solution, amnion products, urinary bladder matrix, and alpha-2 macroglobulin). However, mechanism of action, comparative biological activity, and consistent product formulation to guide informed decisions in treatment selection and disease application are not available in many instances. The role of the innate immune system in inducing and perpetuating chronic low-grade inflammation in multiple disease states (i.e., osteoarthritis) is increasingly recognized and therefore immunomodulation through use of regenerative therapies (e.g., secreted exosomes, cell-cell interactions etc.) represents a therapeutic strategy for further investigation.

With this Research Topic, our goal was to promote a One Health perspective featuring transdisciplinary approaches to improve the understanding of the immunomodulatory role of regenerative therapies in various clinical applications in veterinary medicine or to enhance animal regeneration techniques for human medical research. We aimed to create a Research Topic to share innovative perspectives from specialists across research backgrounds (e.g., immunology, engineering, medicine) to begin to address pressing questions in the field of regenerative therapies. The seven articles in this Research Topic report on a range of therapeutic strategies (e.g., mesenchymal stromal cells, platelet lysate, bone marrow mononuclear cells, senotherapeutics), and further examine the role of the innate immune response in model development of musculoskeletal disease for humans. Below we briefly summarize the main points of each article.

### Mesenchymal stromal cells

In the first article in our series, Cequier et al. evaluated the systemic cellular immune response against equine allogeneic mesenchymal stromal cells (MSC) *in vivo* in horses. Several factors have been shown to influence the interaction of allogeneic MSC with the recipient's immune system, including major histocompatibility (MHC) mismatch or level of MHC expression on MSCs, which may differ following inflammatory licensing or after chondrogenic induction. In this study, naïve MSC, pro-inflammatory primed MSC, or chondrogenically differentiated MSC were repeatedly administered subcutaneously in autologous or allogeneic MHC-matched or MHC-mismatched equine recipients. Increased proliferation of helper and cytotoxic circulating T lymphocytes and IFNγ secretion were observed following administration of MHC-mismatched vs. matched MSCs. Primed MSCs produced the highest immune response, with a similar response after second administration to the first one. These findings indicated that selecting MHC-matched donors is particularly recommended if primed MSC are used or naïve MSC are repeatedly injected, and that matching is less critical for chondrogenically induced MSC.

In the second article of our series, Cassano et al. further evaluated safety of a toll like receptor (TLR)-3 agonist-activated equine allogeneic MSC product injected subconjunctivally three times at 2-week intervals in a pilot study. This study represents a novel route of administration toward further investigation in the context of treating equine recurrent uveitis, the most common cause of glaucoma, cataracts and blindness in horses. Although immunological outcomes were not assessed, no changes in physical examination or evidence of overt systemic inflammation were noted and ocular effects and histopathology were similar between MSC and control eyes. Cellular components (GFP labeled) were found present in conjunctival phagocytic cells. This preliminary safety and tracking information builds upon the body of literature toward implementation of immune conditioned cellular therapies in clinical trials in horses.

### Platelet lysate

In the third and fourth articles in our series, Moellerberndt et al. and Yaneselli et al. examined the impact of platelet lysate (PL) as an alternative to fetal bovine serum (FBS) on the immune characteristics of equine MSC in culture. Moellerberndt et al. reported that PL as a media supplement supported MSC functionality based on cytokine and gene expression, justifying further investigation of functionality of MSC cultured in PL-supplemented media on other immune cell types besides peripheral blood mononuclear cells. Yaneselli et al. examined proliferative capacity, multipotentiality and immune profile of equine MSC following culture, reporting that PL usage led to variation in the immunomodulatory cytokine microenvironment, with elevated IL-6, IL-10, and TNF-α in high-PL concentration media compared to cells cultured in medium PL concentration media or FBS. These findings are topical as replacement of FBS in culture before MSC administration has been proposed to reduce antigenicity and potentially prolong survival after injection and indicate further comparison of functional and immunogenic properties of MSC cultured in alternate serum sources is indicated.

### Alternate regenerative therapies

In the fifth and sixth articles in our series, Everett et al. and Williams et al. describe investigation of alternate therapeutic strategies, focusing on musculoskeletal disorders. Everett et al. reported that intra-articular bone marrow mononuclear cell therapy improves subjective lameness and joint circumference without adverse events in equine naturally occurring osteoarthritis. Williams et al. further expanded upon the potential for senotherapeutics, drug interventions to selectively clear senescent cells, as a promising strategy to prevent or treat multiple age-related conditions in veterinary species. This review article summarized evidence for senotherapeutic activity, preclinical models of disease, ongoing human clinical trials and potential clinical applications in veterinary medicine, providing further justification for studies identifying most active senotherapeutic combinations, dosages and routes of administration for use in veterinary medicine (Williams et al.).

### Immunotherapy to perpetuate model development for human diseases

Finally, in the seventh article in our series, Bonilla et al. examined immunization against nucleus pulposus antigens to accelerate degenerative disc disease in a rabbit model. This study built upon the concept that improved animal models of human and veterinary musculoskeletal disease, such as intervertebral disc degeneration, are needed to comprehensively investigate new diagnostic and therapeutic approaches. This study therefore investigated generation of the active immune response against nucleus pulposus (NP) antigens with an NP vaccine to accelerate and refine the disease model typically triggered by mechanical puncture to the disc, demonstrating that it was possible to elicit an immune response against NP antigens which may contribute to accelerated development of disc disease.

### Summary

The articles summarized here highlight recent advances in our understanding of regenerative therapies and their interaction with the host immune system, which will be valuable to both practicing veterinary clinicians and researchers alike. While further work to evaluate and compare relative functional activity of available therapies is warranted, these studies provide an exciting platform from which further treatments may develop to address disorders that previously limited welfare and performance.

## Author contributions

LP: Writing – original draft, Writing – review & editing. LB: Writing – original draft, Writing – review & editing. LS: Writing – original draft, Writing – review & editing.

